# Understanding the phenotypic spectrum and family experiences of XYY syndrome: Important considerations for genetic counseling

**DOI:** 10.1007/s12687-022-00630-y

**Published:** 2023-01-07

**Authors:** Colleen Jodarski, Rylee Duncan, Erin Torres, Rachel Gore, Armin Raznahan, Morgan Similuk

**Affiliations:** 1 Division of Intramural Research, National Institute of Allergy and Infectious Diseases, National Institutes of Health, Bethesda, MD USA; 2 National Institute of Mental Health, National Institutes of Health, Bethesda, MD USA

**Keywords:** XYY syndrome, Genetic counseling, Neurodevelopment, Psychosocial, Management

## Abstract

XYY syndrome is characterized by a variable neurodevelopmental phenotype, with features including developmental delays, cognitive impairments, and an increased risk for mental health conditions. There are two recent developments that have primarily motivated this review. The first is the increased use of non-invasive prenatal screening (NIPS), which will likely result in more individuals being diagnosed with XYY prenatally. As such, health care providers (HCPs) both within genetics and outside of the specialty are more likely to encounter this diagnosis in the future. The second is advances in the understanding of the phenotypic variability of XYY through biobank and deep phenotyping efforts. As the phenotypic spectrum of XYY syndrome continues to expand, families will face greater uncertainty when receiving this diagnosis. Given both of these developments, HCPs will need to have up-to-date and accurate information about XYY to better counsel families. Furthermore, the ability to employ effective counseling techniques, such as anticipatory guidance, will aid in supporting and guiding families through the diagnostic journey. This review aims to provide insight on the neurodevelopmental and psychosocial aspects of XYY syndrome by discussing current research and borrowing from the relevant psychosocial literature of other genetic conditions. In this way, we hope to equip HCPs with the ultimate goal of improving the care and support provided to individuals with XYY and their families.

## Introduction

XYY syndrome, also known as Jacob’s syndrome, is a sex chromosome aneuploidy (SCA) condition with an estimated prevalence of about 1/1000 male births, though recent studies show that it is underdiagnosed (Abramsky and Chapple 1997; Abramsky et al. 2001; Berglund et al. 2020a, 2019; Nielsen and Wohlert 1991; Zhao et al. 2022). Physical characteristics tend to be less prominent and vary greatly amongst affected individuals but can include tall stature, macroorchidism, macrocephaly, and hypertelorism (Bardsley et al. 2013). While the physical features of this condition tend to be less evident, the neurodevelopmental phenotype is more pronounced and can present challenges for affected individuals and their families.

Historically, most individuals were diagnosed with XYY postnatally after seeking medical care due to concerns such as developmental delay or behavioral issues (Abramsky et al. 2001). This led to early reports of the XYY phenotype being enriched for cognitive and behavioral issues as well as other mental health conditions. While useful in understanding the “severe” end of the spectrum of XYY syndrome, it also created an issue of ascertainment bias within the literature. Recent biobank studies have shed light on the degree of phenotypic variability by providing insight into the “mild to asymptomatic” end of the spectrum. For instance, a recent study of the UK Biobank found that only 1 out of 143 men with 47, XYY had previously known of their abnormal karyotype, further emphasizing the degree to which this condition is underdiagnosed (Zhao et al. 2022).

Additionally, with the advent of non-invasive prenatal screening (NIPS), SCA can now be routinely detected early in pregnancy. Currently, the American College of Obstetricians and Gynecologists (ACOG) recommends that NIPS be offered to all pregnant individuals regardless of age or baseline risk of aneuploidy (ACOG 2020). The increased use of NIPS will likely result in more individuals diagnosed with XYY syndrome in the future, thus increasing the likelihood that health care providers (HCPs) both in the genetics specialty and outside of it will provide care for individuals with XYY. Therefore, it is important for those providing prenatal care to have updated and accurate information to counsel expecting parents and provide appropriate anticipatory guidance. Additionally, pediatric HCPs must better understand the needs of children with XYY and their families to provide them with the necessary resources and care.

Given the recent expansion of the phenotypic spectrum of 47, XYY from large biobank efforts and deep phenotyping of affected individuals, families face the potential of greater uncertainty when receiving a diagnosis of XYY syndrome. Furthermore, with the number of individuals diagnosed with XYY growing due to the increased use of NIPS, HCPs in prenatal and pediatric settings must be equipped to help families navigate the diagnostic journey. As such, the goal of this review is to:

i. Summarize the neurodevelopmental phenotypes of XYY syndrome
ii. Highlight relevant management guidelines
iii. Discuss the individual and family experiences of the diagnostic journey
iv. Provide recommendations for genetic counseling
v. Consider high-priority areas for future clinical research

In doing so, we hope to improve the support provided by the medical community to individuals with XYY and their families.

## Neurodevelopmental features

The neurodevelopmental features of XYY include a mixed picture of language delays, cognitive impairments, and increased risk for mental health conditions. However, the condition is best characterized by marked variability in the presence and severity of the neurodevelopmental phenotype. Individuals may experience developmental delays early in life that persist into childhood and adolescence, while others experience a more subtle phenotype that may go undetected. Here, we outline common cognitive and psychiatric features to increase awareness regarding the neurodevelopmental profile of XYY syndrome.

### Cognitive

Some of the earliest manifestations of XYY syndrome are delays in achieving developmental milestones, including language development and motor skills (Joseph et al. 2018; Lalatta et al. 2012; Ross et al. 2009; Urbanus et al. 2022a, 2022b). Language development delay may be the most characteristic sign in preschool-age children, with marked delay observed in both prenatally and postnatally diagnosed children (Geerts et al. 2003; Urbanus et al. 2022a, 2022b). When compared to individuals with XXY syndrome, one study found that, on average, children with XYY have more severe and pervasive language impairment at both simple and complex levels of oral and written language (Ross et al. 2009). Regarding motor milestones, children with XYY have been noted to have mild delays in the areas of fine motor and gross motor tasks, strength, speed and agility, coordination, and independent walking (Joseph et al. 2018; Ross et al. 2009).

A consistent finding regarding the cognitive phenotype in children with XYY is the wide range in general intelligence (IQ) that trends toward the lower end of normal (Bardsley et al. 2013; Berglund et al. 2020b; S.M. Davis et al. 2020b; Joseph et al. 2018). While both verbal and nonverbal IQ are typically reported in the low-average range, verbal ability tends to be more severely impaired (S.M. Davis et al. 2020b; Joseph et al. 2018; Lee et al. 2012). Individuals with XYY can also have reduced verbal comprehension, working memory, and processing speed, which are associated with increased difficulty maintaining attention and concentration that may result in challenges with other executive functions (Operto et al. 2019).

The speech delay and verbal difficulties observed early in development may contribute to the later onset of learning disabilities (Operto et al. 2019). Academic difficulties among individuals with XYY are common, and individuals often receive special education (Geerts et al. 2003; Linden and Bender 2002). However, it should be noted that individuals with XYY possess other strengths in the academic setting, such as curiosity, humor, and teamwork (Thompson et al. 2022). Additionally, individuals were reported to possess strengths in the areas of science, technology, engineering, and mathematics (Linden and Bender 2002; Thompson et al. 2022).

### Psychiatric

As previously mentioned, individuals with XYY are at an increased risk for mental health conditions (Berglund et al. 2020b). Numerous studies have found that individuals with XYY are more likely than the general population and other SCA conditions to receive a diagnosis of attention deficit-hyperactivity disorder (ADHD) (Bardsley et al. 2013; Kuiper et al. 2021; Ross et al. 2015; Tartaglia et al. 2012). Individuals tend to have greater issues in the domains of inattention and hyperactivity/impulsivity, which may contribute to lower adaptive functioning overall (Bardsley et al. 2013; Joseph et al. 2018; Kuiper et al. 2021; Ross et al. 2015; Tartaglia et al. 2012). Similarly, children with XYY show deficits in both internalizing and externalizing behaviors, with externalizing behaviors such as impulsivity, behavioral regulation, thought and attention problems, and aggression tending to be worse (Fjermestad et al. 2017; Operto et al. 2019; Ross et al. 2012). Although externalizing behaviors are more prominent, some individuals with XYY also show internalizing behavior problems, such as withdrawal (Fjermestad et al. 2017; Lalatta et al. 2012; Operto et al. 2019). Comorbid mood disorders, such as anxiety and depression, tend to be more common in instances where there is a diagnosis of another neurodevelopmental disorder (Bardsley et al. 2013; Tartaglia et al. 2012). Other psychiatric diagnoses reported in individuals with XYY include bipolar disorder and oppositional defiant disorder (Bardsley et al. 2013; Berglund et al. 2020b).

In addition to ADHD, many studies report an increased incidence of autistic behaviors and autism spectrum disorder (ASD) in individuals with XYY compared to the general population and other SCA conditions (Bardsley et al. 2013; Berglund et al. 2020b; Cordeiro et al. 2012; Joseph et al. 2018; Lee et al. 2012; Ross et al. 2012, 2015; Tartaglia et al. 2017). One study reported that individuals with XYY are about five times more likely to have ASD than individuals with XXY and 20 times more likely than the general population (Tartaglia et al. 2017). Importantly, autism symptoms may contribute to social difficulties experienced by individuals with XYY (Cordeiro et al. 2012; Lee et al. 2022, 2012). However, social motivation generally remains intact, suggesting that individuals with XYY are motivated by social interactions, but have trouble in social situations due to social communication deficits and autistic mannerisms (Bouw et al. 2022a, 2022b; Cordeiro et al. 2012; Lee et al. 2022, 2012).

## Management recommendations and multidisciplinary care

There are several management recommendations that were identified during our review of the literature. With respect to developmental delays, caregivers and HCPs should monitor language development to identify any early delays or difficulties (Lalatta et al. 2012; Urbanus et al. 2022a, 2022b). Early identification of language delays allows for early interventions, such as speech therapy, to minimize any long-term detriment (Lalatta et al. 2012; Urbanus et al. 2022a, 2022b). Similarly, it is recommended that children with XYY receive comprehensive neuropsychological and educational screening to evaluate for learning disabilities, ASD, ADHD, and other behavioral or social cognitive difficulties, so that they can receive appropriate interventions, treatments, and services early in development, which allows for improved outcomes (Bouw et al. 2022a, 2022b; Kuiper et al. 2021; Lalatta et al. 2012; Operto et al. 2019; Ross et al. 2012; Tartaglia et al. 2012). In addition to aiding in the development of an individualized education plan for school-aged children, neuropsychological testing may also identify significant impairment in fine motor, gross motor, or self-help skills, in which case physical therapy and/or occupational therapy may be recommended (Thompson et al. 2020; Visootsak and Graham 2006). Additionally, individuals with XYY are at an increased risk of physical comorbidities, such as dystonia and mild tremor, which may require additional care from a neurologist (Bardsley et al. 2013; J.L. Davis et al. 2020a,). Overall, comprehensive screening allows for targeting of each child’s unique needs, and thus, provides the opportunity to create a tailored management plan. In the case of prenatal diagnosis, clinicians should outline these recommendations, so that expecting parents can appropriately plan for follow up, which will aid in bridging the gap between pre- and postnatal care (Lalatta et al. 2012).

The eXtraordinarY Kids Clinic in Colorado presents a model for providing multidisciplinary care to children with SCA (Tartaglia et al. 2015). Their interdisciplinary team consists of developmental-behavioral pediatrics, child psychology, pediatric neuropsychology, speech-language therapy, occupational therapy, genetic counseling, nursing, pediatric endocrinology, and social work (Tartaglia et al. 2015). Such interdisciplinary teams provide the opportunity for comprehensive and individualized care that is tailored to the unique needs of the child. Importantly, parents report that this model of care for their child with SCA improved their understanding of their child, the diagnosis, and the necessary supports to aid in their success (Tartaglia et al. 2015). Given the reported benefits of this care model, implementing similar interdisciplinary clinics in other locations is needed to support the increasing number of individuals diagnosed with SCA as genetic testing implementation continues to expand (Tartaglia et al. 2015).

While early intervention and multidisciplinary care are key to improving outcomes in children with XYY, a recent study also described the importance of interventions that highlight the strengths of children with XYY rather than solely targeting deficits (Thompson et al. 2022). With respect to academic support, strengths-based approaches have been shown to improve student wellbeing, aid academic achievement, and build student capacity (Thompson et al. 2022). Additionally, strength-based approaches in the academic setting help set up students with XYY for success by increasing positive affect, reducing depressive symptoms, and enhancing quality of life (Schutte and Malouff 2019; Seligman et al. 2005). One can easily focus on the potential deficits created by a diagnosis of XYY, but appreciating the strengths and capabilities possessed by these individuals is important as well.

## Individual and family experiences of the diagnostic journey

Whether identified prenatally or postnatally, a diagnosis of XYY can be difficult for families to process. For many families, the diagnosis comes as a surprise, as most parents report not having prior knowledge of SCA at the time of diagnosis (Riggan et al. 2020, 2021). Thus, receiving a diagnosis of XYY can present as a traumatic event and often lead to feelings of grief, distress, and guilt (Lalatta et al. 2012; Riggan et al. 2021). Much of the uncertainty and worry that parents experience at the time of diagnosis can be mitigated by improving their understanding of the diagnosis and its prognosis (Jaramillo et al. 2019; Lalatta et al. 2012; Riggan et al. 2021). While it is common for families to experience feelings of worry and uncertainty, some families, especially those with children diagnosed postnatally, may feel relieved after receiving a diagnosis (Riggan et al. 2020, 2022). Conversely, parents who receive a prenatal diagnosis are at an increased risk of feeling depressed, anxious, and less optimistic (Jaramillo et al. 2019; Riggan et al. 2020, 2021).

In addition to the timing of diagnosis and indication for testing, family experiences of the diagnostic journey can vary based on the information provided during disclosure. For example, parents who report negative experiences at the time of diagnosis often report that their HCP was unprepared, had little information specific to the SCA to share with them, or provided information that they later found was inaccurate, whereas parents with positive diagnostic experiences report that their HCP stressed the phenotypic spectra and connected them with other parents or advocacy groups (Jaramillo et al. 2019; Riggan et al. 2021). The way in which the diagnosis is initially communicated to parents is extremely important because it can influence if additional information is sought and how subsequent information is perceived (Abramsky et al. 2001; Riggan et al. 2021). Despite advances in our understanding of SCA, parents are reporting that they are not presented with materials that are up-to-date and easy to understand at the time of diagnosis (Jaramillo et al. 2019; Riggan et al. 2020, 2021). Another concern is the imbalance of information, as many parents feel that the negative aspects of the condition are emphasized over positive aspects and that the language used was negative or apologetic (Jaramillo et al. 2019; Riggan et al. 2020, 2021). Poor and imbalanced communication at the time of diagnosis may lead families to feel like they do not have the necessary emotional support or information to make informed decisions. As a result, parents may conduct their own internet searches to gather information, which can result in a biased and outdated understanding of XYY (Riggan et al. 2021). HCPs counseling XYY families should be prepared to address any misinformation parents have about the XYY diagnosis and inform them of the limitations in the literature (e.g., institutionalized populations, ascertainment bias, etc.) As access to prenatal genetic screening and testing increases, the likelihood that non-genetics providers will have to disclose results and provide pre- or post-test counseling increases. Since HCPs’ knowledge and beliefs about XYY likely vary significantly, they should be equipped with a disclosure protocol that is based on up-to-date information and follows professional guidelines and input from the XYY community (Abramsky et al. 2001).

As a lifelong diagnosis, parents are continually adapting to what it means to have a child with XYY. Compared to children with XYY, parents perceive their child’s diagnosis as having a larger negative impact on their child’s mental health and well-being, as shown by higher parent-report compared to self-report scores of depression and anxiety (Bardsley et al. 2013). Parents also perceive their children as having greater difficulty in different psychosocial domains compared to how children perceive their challenges (S.M. Davis et al. 2020b). Together, these findings suggest that individuals with XYY may be adapting more positively to their condition than their parents. Parental coping is of particular concern, as reports indicate clinically significant levels of parental stress and anxiety associated with XYY (Lalatta et al. 2012; Operto et al. 2019). While all parents of children with XYY may experience some level of stress, parents of children who have more emotional and behavioral problems (e.g., parents of children diagnosed postnatally) may be of highest concern (Operto et al. 2019). Regardless of the degree of their child’s emotional and behavioral challenges, HCPs must evaluate and monitor parents for signs of stress and anxiety. Additionally, it is important that they acknowledge possible parental anxiety related to a difficult relationship with their child and feelings of inadequacy in aspects of their parental role (Operto et al. 2019).

Following diagnosis, families are commonly faced with the difficult questions of if, when, and how to disclose the XYY diagnosis to their child, other family members, teachers, and care providers. For many families, these decisions are complicated by the variable cognitive, emotional, and behavioral prognoses. Additionally, parents report that their own knowledge and understanding of the condition are barriers to their decision-making (Gratton et al. 2016). Although parents report feeling unsure about how to make decisions about disclosure, research suggests that there are consistent factors upon which parents base their decisions (Gratton et al. 2016). One of the most influential factors is the child’s level of functioning, with parents being more likely to disclose the diagnosis to their child and others if they experience more behavioral, social, and language challenges (Gratton et al. 2016). Although age does not seem to be as important as the child’s functioning, parents were more likely to disclose the diagnosis to older children (Gratton et al. 2016). Many parents of children with XYY must navigate decisions about disclosure on their own, as few received counseling about disclosure at the time of diagnosis (Gratton et al. 2016).

HCPs who interact with children with XYY have an opportunity to support families by helping them navigate the potential benefits and drawbacks of diagnosis disclosure. HCPs should be prepared to address some of the reasons why parents might decide not to disclose, such as fear of stigmatization, feeling like the diagnosis gives an excuse for bad behavior, and needless anxiety if the child is not clinically affected (Gratton et al. 2016). Importantly, part of the counseling discussion regarding disclosure should involve discussion about the variation in outcomes associated with XYY, especially since the child’s functioning plays an important role in the decision-making process. As parents are more likely to disclose the diagnosis if their child is more severely affected, HCPs should be prepared with disclosure resources that are appropriate for all levels of functioning (for examples, see Bishop 2014; Gratton et al. 2014).

## Genetic counseling for XYY

Whether to evaluate the sex chromosomes as part of NIPS has been an area of professional debate due to important considerations on both sides. Reasons one may argue not to screen for SCA include concerns regarding sex selection for non-medical reasons, test inaccuracies, and the less severe and highly variable phenotype of these conditions that result in counseling challenges (Dondorp et al. 2015). For the purposes of this review, we will focus on addressing the latter two issues. At this time, NIPS has a lower positive predictive value (PPV) for SCA compared to trisomies 21, 18, and 13 (Bevilacqua et al. 2018; Guo et al. 2022; Ramdaney et al. 2018). Recent studies report a PPV ranging from 37 to 46% for SCA, which is much lower than the PPV for the other autosomal trisomies with sensitivity and specificity close to 100% (Bevilacqua et al. 2018; Guo et al. 2022; Ramdaney et al. 2018). Additionally, as outlined above, the phenotypic spectrum and severity of XYY are broad with other SCA conditions facing a similar counseling challenge. As such, one may argue to reserve NIPS for only screening for those conditions that have a more severe phenotype. However, in reviewing the literature, parents of children with SCA provide insight into the benefits of early diagnosis.

Firstly, early diagnosis of SCA during pregnancy allows expecting parents more time to prepare and earlier access to recommended therapies and interventions (Bevilacqua et al. 2018; Jaramillo et al. 2019; Riggan et al. 2021). These benefits cannot be understated, as earlier intervention allows for improved outcomes in children with SCA and additional time for parents to adapt to the diagnosis. Additionally, screening for SCA during pregnancy shortens the potential diagnostic odyssey for children who do present with a more severe SCA phenotype. Parents of children with SCA who received a pediatric diagnosis expressed frustration as the early signs of these conditions were missed by medical professionals (Riggan et al. 2022). Furthermore, parents reported that diagnostic delay led to several important negative outcomes for their child, including missed opportunity to access interventions and supports earlier, lack of understanding from others, inability to develop effective parenting strategies at an earlier age, and inability to proactively advocate for their child (Riggan et al. 2022). Given these challenges, one can see how prenatal screening and diagnosis can be of immense benefit to parents in being able to better provide for their child with SCA and adapt to their diagnosis.

Furthermore, studies have shown that current counseling regarding prenatal screening of SCA has room for improvement. For example, parents of children with SCA have reported being unaware of SCA as a possible result from prenatal screening or diagnosis or that a positive NIPS result requires follow up confirmatory testing (Ramdaney et al. 2018; Riggan et al. 2021). Additionally, parents receiving a prenatal diagnosis express a desire for supportive communication concerning parental education and transparency regarding the variable phenotype of SCA conditions (Riggan et al. 2021). Some of the uncertainty of these diagnoses relates to our current understanding of the phenotypic spectrum that will never be fully realized unless SCA is routinely screened for during pregnancy (Berglund et al. 2020a, 2020b). So, while there are currently limitations to screening for SCA with NIPS, we argue that the response to these challenges is not to obscure desired information from expecting parents, but rather to provide accurate and comprehensive pre- and post-test counseling related to SCA.

### Recommendations for clinical practice

The wide variability in the neurodevelopmental phenotype associated with XYY syndrome poses challenges with counseling and providing anticipatory guidance. One of the factors that has been associated with the variability in phenotype is the timing of diagnosis. Individuals who are postnatally diagnosed tend to be more severely affected due to a presenting issue leading them to seek care, whereas individuals diagnosed prenatally are less likely to receive special education or speech therapy services (Bardsley et al. 2013; Lalatta et al. 2012; Linden and Bender 2002). Therefore, the wide variability reported in the prenatally diagnosed population is thought to best reflect the true diversity of the phenotype (Bardsley et al. 2013). As such, HCPs should be flexible in their counseling practices based on timing of diagnosis and indication for testing. Here, we outline recommendations for providing genetic counseling that considers the variability in outcomes and family experiences.

Uncertainty is often a large factor at play within the field of genetics and counseling for XYY syndrome is no different. Given the variability of the phenotype, uncertainty regarding the severity of XYY syndrome, especially in the prenatal setting, is an important concept to discuss with families. The variability in phenotype can also contribute to how well a family is able to adapt to having a child with XYY. For example, Linden and Bender (2002) interviewed families who had a prenatally diagnosed child with XYY and found that while some parents were not concerned about the implications of the diagnosis, others reported concerns about the future welfare of their children and disclosing the diagnosis to others.

When providing information about XYY, HCPs counseling families should strive to offer balanced and accurate information. However, the idea of “balanced” is difficult to define, as shown by relevant literature examining the responses of parents of children with Down syndrome (DS) (Hippman et al. 2012). When asked to provide a “balanced description”, parents provided a wide variety of narratives that likely reflects the differences in their lived experiences with the condition (Hippman et al. 2012). Accordingly, Hippman et al. (2012) provide recommendations for HCP communication that can be strengthened to address challenges related to variability in family experiences. While these recommendations were pulled from the literature on DS, we suggest that they can be utilized when counseling families of children with XYY. HCPs counseling families should recognize the potential variability in a family’s reaction to receiving a diagnosis (Hippman et al. 2012). Regardless of their reaction, exploring previously helpful coping skills can aid in providing anticipatory guidance for families, particularly in the prenatal context (Hippman et al. 2012). Another counseling recommendation is to have expecting parents imagine what their life might be like with an affected child. While XYY is less well known in the general population compared to DS, the lesson still applies, where having parents imagine the similarities and differences of their anticipated life with a child who has XYY can aid in processing the diagnosis and integrating it into the greater framework of their lives.

Additionally, communication surrounding a diagnosis of XYY should be driven by the parent and their specific emotional and informational needs by continuing to ask them for their questions (Hippman et al. 2012). As described above, families are often in need of accurate and up-to-date information about XYY. Awareness of support groups, such as the Association for X and Y Chromosome Variations (AXYS), is a valuable resource for providers and patients alike. Through engaging with the greater community of individuals with XYY, HCPs will be better able to counsel families by providing a nuanced description of the range of possibilities related to life with XYY. So, while “balanced” may be a difficult goal to achieve, providing up-to-date information that is non-judgmental and responsive to the specific needs of the family will be imperative to better counseling families of a child with XYY.

As previously discussed, individuals with XYY are at an increased risk of developing certain psychiatric conditions. While not all children with XYY will develop psychiatric issues, it is important to equip parents to identify symptoms so that they can better manage their child’s mental health. Drawing from the lessons learned from 22q11.2 deletion syndrome (22qDS) and “awareness to act”, HCPs can empower parents of children with XYY to be their child’s best advocate. Carrion et al. (2022) define “awareness to act” as confidence in being alert and equipped to protect and/or manage their child’s mental health. Parents of children with 22qDS described worry and stress over missing “red flags” of mental illness and a limited awareness as to what symptoms they should be watching out for (Carrion et al. 2022). One can easily see how this could apply to other conditions that carry a risk of psychiatric conditions, which are more difficult to screen for at times than physical symptoms. Parents in this study reported that psychiatric genetic counseling helped them achieve awareness to act in several important domains: increased confidence in awareness of red flags, strategies to protect their child’s mental health, and empowerment in their ability to articulate and advocate for services their child needed (Carrion et al. 2022). Most importantly, providing anticipatory guidance promoted parents’ sense of agency in reducing the risk of psychiatric problems and improving outcomes for their child with 22qDS (Carrion et al. 2022). Since parents of children with XYY may experience a great deal of stress related to their child’s diagnosis and perceived mental health, they may also benefit from a similar form of anticipatory guidance. Given that most of the presentations of XYY are “unseen” physically, empowering families in a similar way is important so that they can advocate effectively for their child’s mental health needs.

## Areas for future research

While this is not an exhaustive list of areas for of future research, our review of the literature identified significant gaps in our current understanding of XYY syndrome and its impact on individuals and their families. Firstly, future research should focus on qualitative reports from the families themselves about their experiences of having a child with XYY. In this way, we can learn what is most helpful and most challenging about living with XYY as well as identify family needs that are currently unmet by HCPs. Furthermore, most of the referenced studies include data obtained from parental report, which at times was shown to differ from what was reported by individuals with XYY (Bardsley et al. 2013; S.M. Davis et al. 2020b). Future research should also focus on the experiences of individuals with XYY to gauge their understanding of the diagnosis and what their specific needs are.

Additionally, more longitudinal studies, such as the eXtraordinarY babies study, are needed to enhance our understanding of XYY across the lifespan (Tartaglia et al. 2020). Similarly, there are few studies to date about the impacts of an XYY diagnosis in adulthood and the challenges that may occur during this transition. The transition to adulthood marks a time of great change and uncertainty, and better understanding of how individuals with XYY adapt during this time is essential. In this way, HCPs can provide appropriate support and counseling regarding specific concerns, such as fertility and family planning.

Future work should also aim to distinguish possible biological and familial predictors of phenotypic variability. For example, multiple linear regression models have shown that individuals with XYY and their family members are correlated on certain traits, such as IQ, vocabulary, and social awareness (Wilson et al. 2021). Efforts to understand the clinical utility of these models in the genetic counseling setting will be useful in providing anticipatory guidance for families. As such, HCPs will be able to better counsel families about the issues surrounding diagnosis, both medically and psychosocially.

## Conclusion

In conclusion, XYY syndrome is characterized by a neurodevelopmental phenotype that is variable in nature. Given that the primary manifestations of this condition are largely ‘unseen’, individuals and their families must navigate unique challenges, such as disclosure of diagnosis. An XYY diagnosis can lead to a range of experiences and emotions for family members, with parental coping being of particular concern. As such, anticipatory guidance and preparing families with an “awareness to act” are useful counseling interventions. Additionally, it is vital that families be provided with resources about the diagnosis that are developmentally appropriate for their child. There is room to grow in our understanding of the many facets of an XYY diagnosis and the associated psychosocial challenges. With future research focusing on the phenotypic spectrum of this condition across the lifespan, special attention will also need to be paid to how this diagnosis also impacts the family system. XYY syndrome is a diagnosis that has significant implications on not only the affected individual, but the family as well. It is therefore imperative that HCPs fully understand the range of lived experiences with XYY so that we can better counsel and care for affected individuals and their families.
