# Correction to: Understanding the phenotypic spectrum and family experiences of XYY syndrome: Important considerations for genetic counseling

**DOI:** 10.1007/s12687-023-00636-0

**Published:** 2023-02-10

**Authors:** Colleen Jodarski, Rylee Duncan, Erin Torres, Rachel Gore, Armin Raznahan, Morgan Similuk

**Affiliations:** 1 Division of Intramural Research, National Institute of Allergy and Infectious Diseases, National Institutes of Health, Bethesda, MD USA; 2 National Institute of Mental Health, National Institutes of Health, Bethesda, MD USA

**Correction to: Journal of Community Genetics**

10.1007/s12687-022-00630-y

The original version of this article unfortunately contains mistakes.

The funding statement should read: “This work was supported with funds from the NIAID Division of Intramural Research and from the National Institute of Mental Health (NIMH).”

This is being corrected in this publication.

